# Time to initiation of modern contraceptive method use after childbirth and its predictors in Southern Ethiopia: a retrospective follow-up study

**DOI:** 10.1186/s12905-023-02809-y

**Published:** 2023-12-08

**Authors:** Erjabo Adinew Mugoro, Dejene Ermias Mekango, Tigist Alebachew Lule, Belayneh Hamdela Jena, Ermias Abera Turuse

**Affiliations:** 1 Department of public health, school of public health, college of medicine and health sciences, Wachemo University, Hossana, Ethiopia; 2 Department of reproductive health, school of public health, college of medicine and health sciences, Wachemo University, Hossana, Ethiopia; 3 Maternal and child health care unit, Wachemo University Nigist Eleni Mohammed Memorial Hospital, Wachemo University, Hossana, Ethiopia; 4 Department of epidemiology and biostatistics, school of public health, college of medicine and health sciences, Wachemo University, Hossana, Ethiopia

**Keywords:** Median time, Predictors, Modern contraceptive use, Postpartum women

## Abstract

**Background:**

Timely initiation of modern contraceptive use is vital to prevent unintended pregnancy and its related morbidities and mortalities. However, there is a scarcity of evidence about the duration of time elapsing from childbirth to initiating modern contraceptive use following childbirth and its associated factors in the study area for evidence-based interventions. Therefore, we aimed to assess the time to initiation of modern contraceptive method use and its predictors in Hossana town, southern Ethiopia.

**Methods:**

A retrospective follow-up study was conducted at public health facilities in Hosanna town. A total of 503 study participants were included in the study using a systematic random sampling technique. The Box and Whisker plot was used to estimate the time to initiation of modern contraceptive use. A Weibull regression model was applied to identify predictors of time to initiation of modern contraceptive use. Adjusted Hazard Ratio (AHR) with a 95% confidence interval (CI) was used to interpret the strength of the association.

**Results:**

The median time to initiation of modern contraceptive use was 6 months, with an interquartile range of 3 months. Husband/partner attending higher education [AHR = 1.64, 95% CI: 1.04, 2.57], women who had parity of more than two [AHR = 1.93, 95% CI: 1.01, 3.67], and women who had communicated with their husband/partner about modern contraceptive methods [AHR = 3.03, 95% CI: 1.41, 6.67] were more likely to initiate modern contraceptive method use within six months after childbirth. In contrast, women with an older age of greater than or equal to 30 years [AHR = 0.32, 95% CI: 0.13, 0.82] and who did not resume sexual intercourse after childbirth [AHR = 0.02, 95% CI: 0.01, 0.03] were less likely to initiate modern contraceptive method use within six months after childbirth.

**Conclusions:**

The median time to initiation of modern contraceptive method use after childbirth in the postpartum period was delayed from the World Health Organization recommendation of at most 6 weeks. Emphasis should be given to older women, women with lower parity, and men’s participation in contraceptive communication to improve timing for initiation of modern contraceptive use after childbirth and to curb the five-month lag periods.

**Supplementary Information:**

The online version contains supplementary material available at 10.1186/s12905-023-02809-y.

## Background

The time elapsed from childbirth until initiation of modern contraceptive use is crucial to prevent unintended pregnancy and its consequences, such as unsafe abortion, hemorrhage, sepsis, and maternal death [1]. Timely initiation of postpartum modern contraceptive use (in the first month of childbirth) plays an important role in strategies to achieve adequate intervals between pregnancies (at least 2 years) [2]. More than 30% of maternal deaths and 10% of child deaths can be prevented by spacing pregnancy for at least 2 years using modern contraceptive methods. Thus, timely initiation of modern contraceptive method use after childbirth saves the lives of mothers and children [3].

Globally, in 2019, the proportion of family planning requirements met by modern methods was 75.7%. However, demographic and health survey data analysis from 27 countries shows that 95% of women who are 0–12 months postpartum want to avoid a pregnancy in the next 2 years, but 70% of them are not using contraception [4]. A significant proportion of women, particularly in developing countries, have sexual intercourse after childbirth without any form of contraception [5]. Empirical studies revealed that women in low and middle income countries including Ethiopia, less utilize modern methods often due to lack of decision-making autonomy, perceived social norms (approval of or conformability to others), fear of side effects, myths and misconceptions [6, 7]. In Sub-Saharan Africa, nearly one-third of postpartum women are at risk of becoming pregnant from unprotected sex within the first two years of delivery [8].

In Ethiopia, even in contraceptive-accessible areas like urban areas, a sizable proportion (50%) of women did not use any form of modern method. Still, 22% of women have an unmet need for modern contraception (13% for spacing and 9% for limiting) [9]. Many postpartum women in Ethiopia resumed sexual intercourse without contraception. For instance, a study in Ethiopia found that 78.3% of postpartum women resumed intercourse after giving birth, with a median time of 6 weeks with a late start to contraception [10]. According to the studies in Gondar city and Gozamen district, the median time to use modern contraceptives among women during the extended postpartum period was 6 and 3.2 months, respectively [11, 12]. In Ethiopia, it is common for women to have too many children who are too close in age. As a result, the country’s population has increased significantly, but economic growth has not kept pace. An unbalanced population will inevitably have a negative impact on the nation’s well-being. Modern contraception is one of the strategies that is proving to be effective in addressing these issues [12].

According to the World Health Organization medical eligibility criteria for contraceptive use, women should be offered contraceptives within the first month of postpartum [13]. As all nations also share, the Ethiopian government has an ambition to reduce current maternal mortality (412 per 100,000 live births) to at least 70 per 100,000 live births at the end of 2030 [14]. To achieve this sustainable development goal, timely initiation of modern contraceptive method use is one of the key strategies, globally [15].

Therefore, we aimed to assess the duration of time to initiate modern contraceptive use and its predictors among postpartum women visiting public health facilities for child immunization in Hossana town, southern Ethiopia. The finding will contribute to improving maternal and child health by reducing unintended pregnancies and related morbidities and mortalities at local and national levels.

## Methods

### Study design and setting

A health facility-based retrospective follow-up study was applied to this study. The study was conducted in Hossana town, which is found in Hadiya Zone, southern Ethiopia. Hossana town is located 232 km from the capital city, Addis Ababa, and 294 km from its regional capital city, Hawassa. There is one comprehensive specialized hospital and three health centers (Hosanna, Lichamba, and Bobicho) that offer maternal and child health care services [Hadiya Zone Health Department, unpublished report]. The study was conducted in these health facilities from May 25 to July 25, 2021.

### Participants

For this study, women in an extended postpartum period with a one-year-old child who were attending public health facilities for their child’s vaccination during the study period (May 25 to July 25, 2021) were included and retrospectively followed to get data.

### Variables and measurements

The dependent variable was the time spent from childbirth to initiation of modern contraceptive use in completed months. A follow-up period was defined as the number of months between a childbirth and the initiation of modern contraceptive use. The event was the initiation of modern contraceptive use in the first six months, whereas the censored was not initiating modern contraceptive use within six months of childbirth. The independent variables were defined as follows: Residence: refers to where the women usually live, categorized as urban or rural.

Education status: refers to the level of education that an individual attended, categorized as no formal education (no grade), primary (1-8th grade), secondary (9-12th grade), higher (attending colleges and Universities).

Occupation: refers to the main job that an individual works.

Parity: the number of times that a woman has given birth, irrespective of outcome.

Family planning council during ANC visits: refers to whether a woman received advice about modern contraceptive use after delivery or not, categorized as “yes” if received or “no” otherwise.

Future reproductive plan: refers to whether the couples have a plan to have additional children or not in the future, categorized as wanting no more children, wanting children within two years, wanting children at and after two years, and undecided.

Communication with partner about modern contraceptive methods: refers to whether a woman has had any discussion about modern contraceptive method use after delivery with her husband or not, categorized as “yes” or “no.“

Husband approval: refers to whether the husband agree or support his wife to use modern contraceptive methods or not, responded as “yes” if agreed and “no” otherwise.

Decision-maker for contraceptive use: measured by asking a woman that who would make a decision to use modern contraceptive methods when needed, categorized as “wife alone,“ “jointly,“ or “husband.“

Return of menses: whether a woman has seen her menses before initiating contraceptive use or not, responded as “yes” or “no”.

Resumption of sexual intercourse: refers to whether the couples, after recent childbirth, have had initiated sexual intercourse or not before using contraceptive methods, responded as “yes” or “no”.

### Sample size and sampling procedure

The sample size was determined using Epi info version 7 for proportional hazard regression model by taking 80% power, 5% level of significance, 0.5 standard deviation, 95% confidence interval (CI), and %outcome in the unexposed group of 22.83, %outcome in the exposed group of 35%, and AHR of 1.53. [16]. Finally, the total sample size with 10% non-response rate was 513.

To select the study participants, a systematic random sampling technique was applied in each health center and hospital. The last six-month immunization services for children in each health facility were considered to estimate the number of postpartum women visiting the health facilities (sample frame) and calculate the sample fraction. The total sample size (513) was proportionally allocated to each health facility (Hossana health center = 181, Wachemo University Nigist Eleni Mohammed Memorial hospital = 127, Lichamba health center = 110, and Bobicho health center = 95). Based on the calculated sample fraction of each health facility (k = 8), the study participants were included in the study. The first participant was selected using a simple random sampling technique (lottery method).

### Data collection tools and procedures

Data were collected using structured interviewer administered questionnaire adapted from previously done similar researches and modified according to the local context by the investigators. The questionnaire was prepared in English, then translated into Amharic and back into English by experts in both languages to check its consistency. The pre-test was carried out on 5% of the sample size prior to the actual data collection time in Homecho Hospital to make necessary adjustments after obtaining informed consent. The questionnaire was checked for its clarity, understanding ability, uniformity, and completeness of the questions. Necessary amendments, such as skipping and changing the order of questions, were made based on the pre-test result.

A structured interview questionnaire and chart review were used to obtain background information or potential predictors affecting time to modern contraceptive use, such as sociodemographic and economic factors, pregnancy history, reproductive characteristics, access to service & health information related and decision-making, Data collectors (four BSc midwives) who are familiar with the local language and supervisors (four public health professionals) participated in data collection after receiving two days of training by the principal investigator about the data collection instrument, ethical considerations, and objectives of the study.

### Data processing and analysis

Data were coded and entered into Epi-data version 7 software and exported to Stata version 14 for the analysis. Before the analysis, the data were checked for missing values and outliers. For quantitative variables means, median and standard deviations were calculated. Continuous variables were categorized using Ethiopian demographic and health survey reports and recoding was done when the cells are few in number. For categorical variables, descriptive statistics such as frequencies and percentages were calculated. The Box and Whisker plot was used to compute the median survival time (time to initiation of modern contraceptive use in months). A cox-proportional hazard assumption was checked using a Schenofeld residual test (global test), and the proportionality assumption was violated. As a result, a parametric model was used as an option, and a parametric distribution that fits the data reasonably better (the Weibull distribution) was selected using a log-likelihood in the null model. In the bivariable Weibull regression model, variables that have shown a significant association with time to initiation of modern contraceptive method use at *P* < 0.25 were included in the multivariable model. In the multivariable Weibull regression model, variables with a 95% CI for the adjusted hazard ratio that did not include 1 and a *P* < 0.05 were declared as predictors of time to initiation of modern contraceptive method use.

### Data quality control measures

Data quality was assured before, during, and after the data collection process. Before data collection: structured questionnaire was developed from different literature conducted to collect data. It was translated from English to Amharic and back to English to assure consistency. In addition, questionnaire was pre-tested, and training was given for data collectors and supervisors. During data collection, there was close day-to-day supervision. The collected data were checked for completeness and consistency by the supervisors and principal investigator. Adequate time (until they respond) was given for the participants to remember the date variables (age, time of contraceptive use, resumption of sexual intercourse, return of menses) to minimize recall bias. After data collection, the supervisors and the principal investigator together rechecked the completeness and consistency before transferring it into computer software. Non-overlapping numerical codes were given for each question, and then coded data was entered into Epi-data version 7.

## Results

### Socio-demographic and other characteristics

Of 513 women with one-year-old children who came to the immunization unit, 503 were successfully interviewed, yielding a response rate of 98.05%. The mean age of the study participants was 29.8 ± 5.6 years. The ages range from 17 to 44 years. Three hundred sixty five (72.6%) of the women were protestant religion follower (Table 1).

**Table 1:** Socio-demographic and other characteristics of the study participants in Hossana town, Southern Ethiopia, 2021

Variables	Frequency (%)
Marital status	
Married	482 (95.8)
Single	21 (4.2)
Ethnicity	
Hadiya	339 (67.4)
Kembata/Tembaro	85 (16.9)
Amhara	30 (6)
Guragie	23 (4.6)
Siltie	15 (3)
Others^*^	11 (2.2)
Religion	
Protestant	363 (72.2)
Orthodox	76 (15.1)
Catholic	35 (7)
Muslim	27 (5.4)
Others^**^	2 (0.4)
Maternal occupation	
Housewife	264 (52.5)
Employed	175 (34.8)
Merchant	22 (4.4)
Student	42 (8.3)
Husband/partner occupation	
Employed	338 (67.2)
Merchant	76 (15.1)
Farmer	89 (17.7)
Antenatal care visit during the recent pregnancy	
Yes	499 (99.2)
No	4 (0.8)
Postnatal care visit after the recent childbirth	
Yes	95 (18.9)
No	408 (81.1)

Keys: * = Wolayita, Oromo, Tigrie. **= Adventist, apoplectics

### Time to initiation of modern contraceptive use

**Fig. 1:**
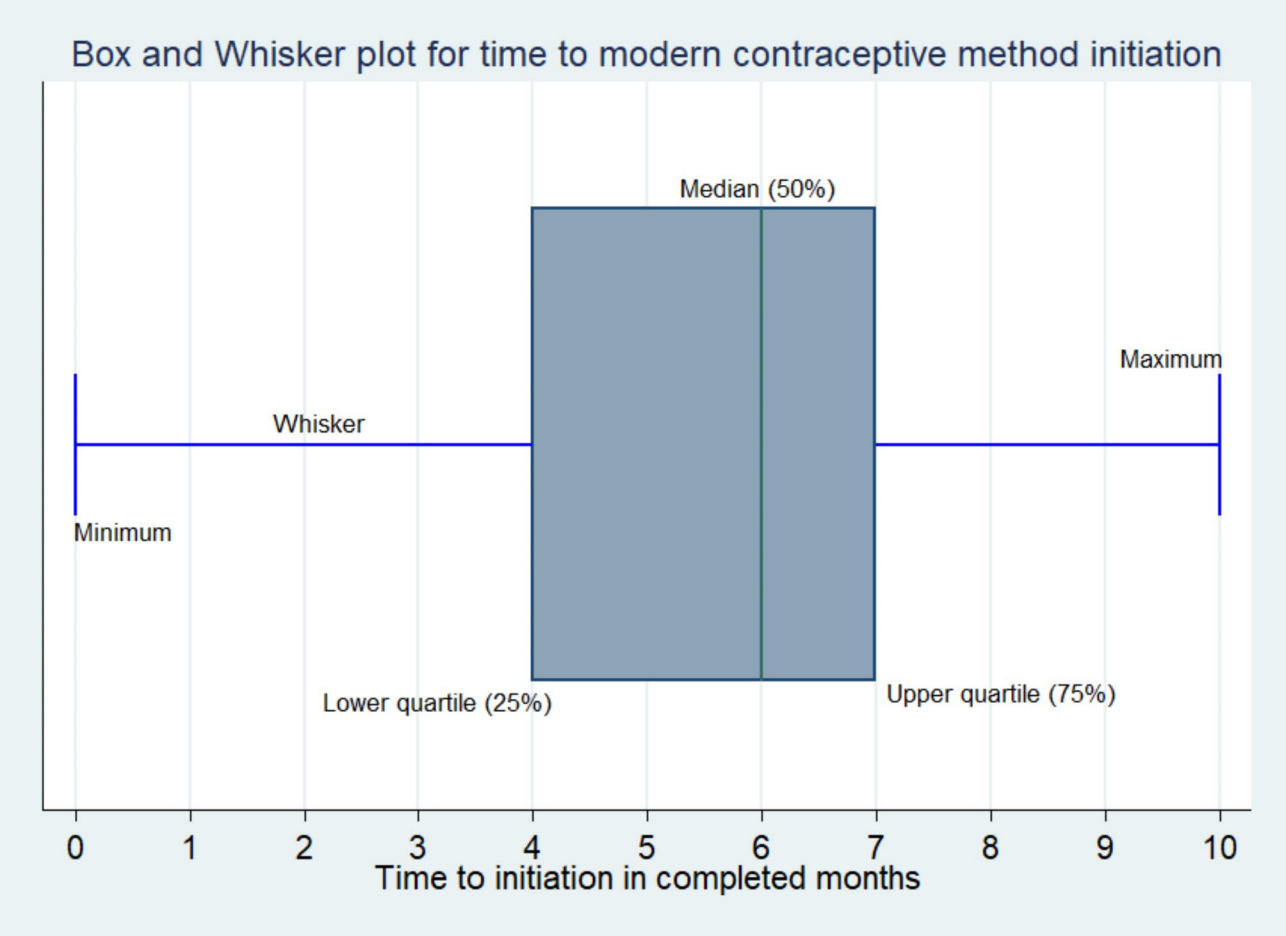
Cumulative survival time to initiation of modern contraceptive method use in completed months among study participants in Hossana town, Southern Ethiopia, 2021

The median survival time to initiate modern contraceptive method use among postpartum women was 6 months, with an interquartile range of 3 months. The proportion of “events” (women who initiated modern contraceptive method use within six months) was 217 (43.1%), and the proportion of “censored” (women who did not initiate using the modern contraceptive methods within 6 months) was 286 (56.9%). Of 217 women who had initiated modern contraceptive methods, 10 (4.6%) pill (progestin only pill), 114 (52.7%) injectable, 90 (41.4%) implants, and 3 (1.3%) male condom users. The cumulative probability of surviving (not initiating modern contraceptive method use) was decreasing as months in the postpartum period increased: 88% within the first month, 80% at the third month, and 57% at the sixth month (Fig. 1).

### Predictors of time to initiation of modern contraceptive use

The Cox proportional hazard assumption was checked for the covariates using a Schoenfeld residual test, and the proportionality assumption was violated (Global test chi-square = 45.08, df = 13, and Prob > chi2 = 0.0000) (Additional file 1). Thus, fitting a suitable parametric survival model that provides a hazard ratio (distributions that report log relative hazard) for better interpretation was chosen. Accordingly, we compared three parametric distributions (Exponential, Gompertz, and Weibull) using a log-likelihood in the null model to select a distribution that fit the data reasonably better. The Weibull distribution found to provide a higher log-likelihood (-362.94) than Exponential (-390.95), and Gompertz (-379.53), and selected as better distribution for our data.

In a bivariable Weibull regression model, maternal age, residence, maternal education, husband’s education, parity, number of alive children, family planning counseling during the recent antenatal care, the future reproductive plan, communication between partners, husband approval, decision-making about contraception, return of menses, and resumption of sexual intercourse were significantly associated with time to initiation of modern contraceptive method use at *P* *<* 0.25.

In multivariable Weibull regression model, maternal age ≥30 years, husband’s attending a higher education, parity of more than two, communication with husband about modern contraceptive methods, and resumption of sexual intercourse were found to be statistically significant predictors of time to initiation of modern contraceptive method use, with a 95% confidence level and *P* *<* 0.05. Women attending secondary education (9-12th grade) and return of menses were marginally significant predictors.

Accordingly, women who were 30 years and older were 68% [AHR = 0.32, 95% CI: 0.13, 0.82] less likely to initiate modern contraceptive methods within six months after childbirth than women who were 17–24 years old. Husband/partner who attended a higher education were nearly twice [AHR = 1.64, 95% CI: 1.04, 2.57] more likely to initiate modern contraceptive methods within six months than those attending a primary and lower education. Women who had parity of more than two were nearly twice [AHR = 1.93, 95% CI: 1.01, 3.67] more likely to initiate modern contraceptive methods within six months than para two and lower. Women who have communicated about modern contraceptive methods with their husband/partner were three times [AHR = 3.03, 95% CI: 1.41, 6.67] more likely to initiate modern contraceptive methods within six months as compared to their counterparts. Women who had not resumed sexual intercourse after the recent childbirth were 98% [AHR = 0.02, 95% CI: 0.01, 0.03] less likely to initiate modern contraceptive methods within six months than those who had resumed sexual intercourse (Table 2).

**Table 2:** Multivariable Weibull regression model for the predictors of time to initiation of modern contraceptive method use in public health facilities of Hossana town, Southern Ethiopia, 2021

Variables	Time of modern contraceptive initiation		CHR (95% CI)	AHR (95% CI)
	Event (< 6 months)	Censored (≥ 6 months)		
Maternal age in years				
17–24	52 (50.5)	51 (49.5)	1	1
25–29	73 (45.6)	87 (54.4)	0.69 (0.46, 1.06)°	0.63 (0.31, 1.27)
≥ 30	92 (38.3)	148 (61.7)	0.62 (0.42, 0.92)^∗^	0.32 (0.13, 0.82)^*^
Residence				
Urban	134 (49.6)	136 (50.4)	1.69 (1.23, 2.34)^**^	1.31 (0.93, 1.86)
Rural	83 (35.6)	150 (64.4)	1	1
Maternal education				
Zero-primary	53 (27.6)	139 (72.4)	1	1
Secondary	94 (49.2)	97 (50.8)	2.22 (1.52, 3.24)^***^	1.46 (0.95, 2.23)
Higher	70 (58.3)	50 (41.7)	2.29 (1.48, 3.52)^***^	1.01 (0.62, 1.65)
Husband’s education				
Zero-primary	63 (36)	112 (64)	1	1
Secondary	83 (40.7)	121 (59.3)	1.34 (0.91, 1.97)°	1.24 (0.83, 1.86)
Higher	71 (57.3)	53 (42.7)	2.02 (1.35, 3.04)^**^	1.64 (1.04, 2.57)^*^
Parity				
1–2	117 (45.9)	138 (54.1)	1	1
> 2	100 (40.3)	148 (59.7)	0.91 (0.66, 1.24)^°^	1.93 (1.01, 3.67)^*^
Number of alive children				
1	60 (53.1)	53 (46.9)	1	1
2–3	117 (42.4)	159 (57.6)	0.63 (0.44, 0.89)^*^	0.79 (0.38, 1.65)
≥ 4	40 (35.1)	74 (64.9)	0.49 (0.31, 0.78)^**^	0.95 (0.37, 2.42)
Family planning counseling during the recent antenatal care visits				
Yes	83 (55.7)	66 (44.3)	1	1
No	134 (37.9)	220 (62.1)	0.57 (0.41, 0.78)^**^	0.92 (0.64, 1.34)
Future reproductive plan				
Undecided	10 (21.3)	37 (78.7)	1	1
Want children within 2 years	160 (48)	173 (52)	3.08 (1.43, 6.60)^**^	1.63 (0.72, 3.72)
Want children at or after 2 years	36 (43.9)	46 (56.1)	2.98 (1.31, 6.79)^**^	1.19 (0.48, 2.91)
Want no more child	11 (26.8)	30 (73.2)	1.27 (0.45, 3.62)	1.45 (0.48, 4.34)
Communication with husband/partner about modern contraceptive methods				
Yes	203 (51.3)	193 (48.7)	5.62 (2.94, 11.11)^***^	3.03 (1.41, 6.67)^**^
No	14 (13.1)	93 (86.9)	1	1
Husband approval				
Yes	128 (55.9)	101 (44.1)	1	1
No	89 (32.5)	185 (67.5)	0.51 (0.37, 0.69)^***^	0.44 (0.07, 2.79)
Decision-maker for modern contraceptive method use				
Wife	5 (17.9)	23 (82.1)	1	1
Jointly	126 (55.8)	100 (44.2)	3.07 (1.25, 7.55)^*^	0.92 (0.21, 4.01)
Husband/partner	86 (34.5)	163 (65.5)	1.69 (0.68, 4.21)	1.78 (0.53, 5.96)
Return of menses				
Yes	104 (48.1)	112 (51.9)	1	1
No	113 (39.4)	174 (60.6)	0.38 (0.27, 0.52)^***^	1.40 (0.96, 2.04)
Resumption of sexual intercourse				
Yes	140 (88.6)	18 (11.4)	1	1
No	77 (22.3)	268 (77.7)	0.02 (0.01, 0.03)^***^	0.02 (0.01, 0.03)^***^

Keys: Significant *** = P < 0.001, ** = P < 0.01, * = P < 0.05. ^*°*^ *= P < 0.25. CHR: Crude Hazard Ratio. AHR: Adjusted Hazard Ratio CI: Confidence Interval. 1 = reference category*

## Discussions

This study aimed to assess the median time to initiation of modern contraceptive method use after childbirth and its predictors among postpartum women. Accordingly, the median time to initiation of modern contraceptive method use was six months, which is associated with maternal age, husband/partner education, parity, communication with husband/partner about contraceptive methods, and resumption of sexual intercourse after childbirth.

In this study, the median time to initiation of modern contraceptive method use after childbirth was six months. The finding of this study suggests that there is a greater delay in initiating contraceptive use after childbirth than recommended by the World Health Organization, which is six weeks [13]. Women in this period could be prone to unintended pregnancy and its consequences, especially when their menses has returned. In Sub-Saharan Africa, including Ethiopia, the rate of mistimed pregnancies is becoming a problem [17–20]. Approximately half of all pregnancies reported to have come soon, which could have been prevented with increased access to effective utilization of modern postpartum contraceptive methods in a timely manner [17]. The finding of this study was consistent with that of Gondar City, Northwest Ethiopia, which was 6 months [11], and Ambo Town, Central Ethiopia, which was 6 months [21]. The finding was a bit higher than the finding from the national report of four months [22], but lower than the median time reported from Uganda, 19 months [23]. The differences could be due to variation in awareness, access, and availability of modern contraceptive methods, desire for the number of children, decision-making autonomy for contraceptive methods, sample size, the study population considered, and other socio-cultural issues among the countries or study settings.

In this study, women with an older age of 30 or more years were less likely to initiate modern contraceptive method use after childbirth than those with an age of 17 to 24 months. The finding highlights a need for attention for those older women. As women ages goes to older and older (such as ≥30 years), fertility usually decline, and women might have few reproductive times to reach desired number of children [24]. As a result, they might be less likely to use modern contraceptive methods. On the other side, they might have experience of using traditional methods, particularly if they have a regular cycle, so that they less intend to utilize the modern methods [25]. The finding was supported by the findings from Ambo Town and Myanmar, which revealed that older women were less likely to initiate the modern contraceptive method use than those with younger ages [21, 26, 27].

Women with a parity of more than two were more likely to initiate modern contraceptive method use than those with a parity of one to two. Those women with parity of more than two might have reached the desired number of children sooner than those with lower parity. Hence, they may want to space or limit the number of children by using modern contraceptive methods. A study from Ambo Town reported the contrary finding that women with higher parity were less likely to initiate modern contraceptive use following childbirth than those with lower parity [21]. This could be due to socio-cultural variation in the desired number of children in different parts of the country. In some societies, a number of children believed to be an asset for family, as they believed to support parents/families in different circumstances such as income generation, sense of security or protection of family, including females, ease of labour, etc. [28].

This study found that husband/partner with higher education were more likely to initiate modern contraceptive use than those with primary or lower educational attainment. This indicates that an educated husband/partner might have supported or encouraged his wife to use modern contraceptive methods after childbirth. Higher educated husband/partner might have a better access to health care information, and greater ability to use health care services, increases their discussion about the methods than those who had lower education level [29, 30].

Communication of contraceptive issues with their husband/partner significantly associated with timing of modern contraceptives method initiation. The finding suggests the importance of men’s involvement in maternal health care service utilization, such as modern contraceptive use. Communication between couples might have improved the confidence of women to utilize contraceptive services in time. The finding was supported by the study done in northern Ethiopia, Senegal, and Niger that showed couples’ communication about modern contraception was significantly associated with improved utilization of modern contraceptive methods [31–33].

Resumption of sexual intercourse after childbirth was found to be an inducing factor to initiate modern contraceptive method use. This could be due to the couple’s awareness of the risk of getting pregnant, possibly an unintended pregnancy, and other related issues such as poor maternal and child health status due to inadequate spacing. The finding was supported by the other studies [34, 35].

Despite the attempts made to reduce bias, this study might have limitations: the study relied on the ability to recall the time of initiation of modern contraceptive method use, and the first sexual initiation, which were difficult to remember accurately. Moreover, this study relied on women bringing a one-year-old child to health facilities for vaccination, and some women who did not bring their children for vaccination were not included in the sample. Furthermore, some women who had a one-year-old child but are pregnant might not have been included in the study, as they might not have visited vaccination clinics. As a result, selection bias might have occurred to some extent. Despite the limitations, the findings can be generalized for population with similar contexts.

## Conclusions

The median time to initiation of modern contraceptive method use after childbirth in the postpartum period was delayed from the World Health Organization recommendation of at most 6 weeks. Emphasis should be given for older women, women with lower parity, and men participation in contraceptive communication to improve time to initiation of modern contraceptive use after childbirth, and to curb down the five month lag periods. Improving clients’ awareness of the advantages of timely initiation of modern contraceptive methods, such as the prevention of unintended pregnancies, reducing the risk of abortion, preventing pregnancy-breastfeeding overlaps, preventing maternal nutritional depletion, preventing low birth weight, etc., is crucial.

### Electronic supplementary material

Below is the link to the electronic supplementary material.

Supplementary Material 1

Supplementary Material 2

## Data Availability

The raw materials that support the conclusions of this research will be available to researchers, who need the data to use for non-commercial purposes through requesting the corresponding author.
