# Supplementary figures and images for: Time to initiation of modern contraceptive method use after childbirth and its predictors in Southern Ethiopia: a retrospective follow-up study
Source: BMC Womens Health. 2023 Dec 8;23:658. doi: 10.1186/s12905-023-02809-y (PMC10704612; doi:10.1186/s12905-023-02809-y)

**Additional file 2. Kaplan-Meir curve for the predictors**


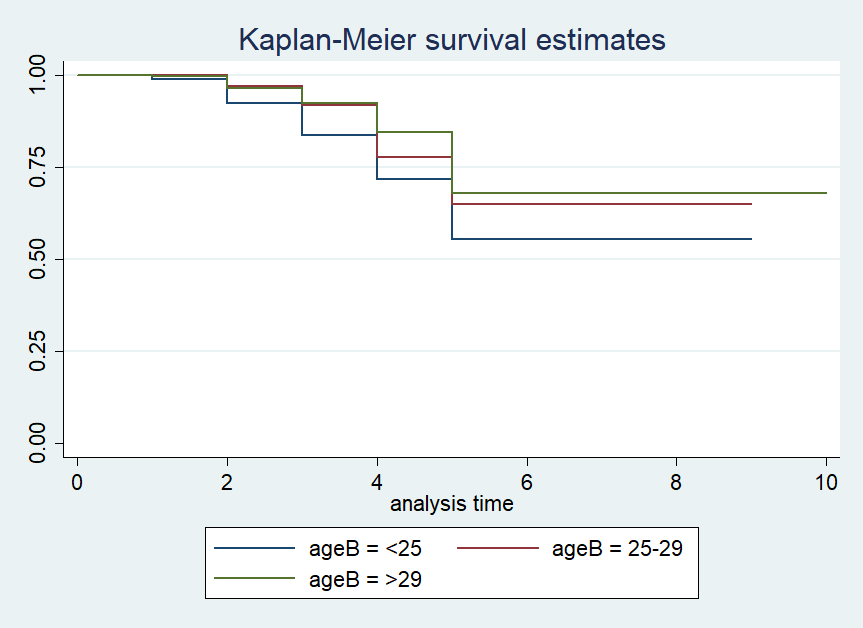


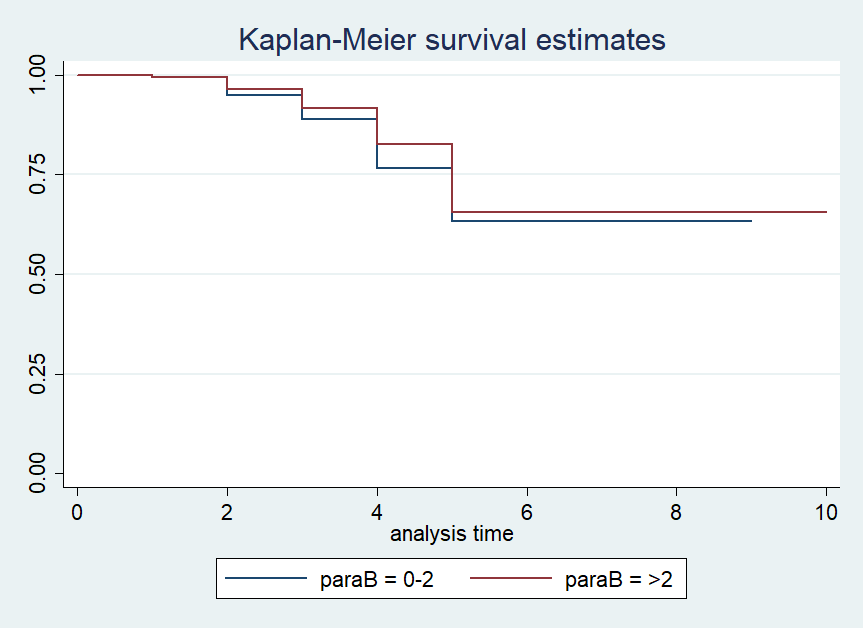


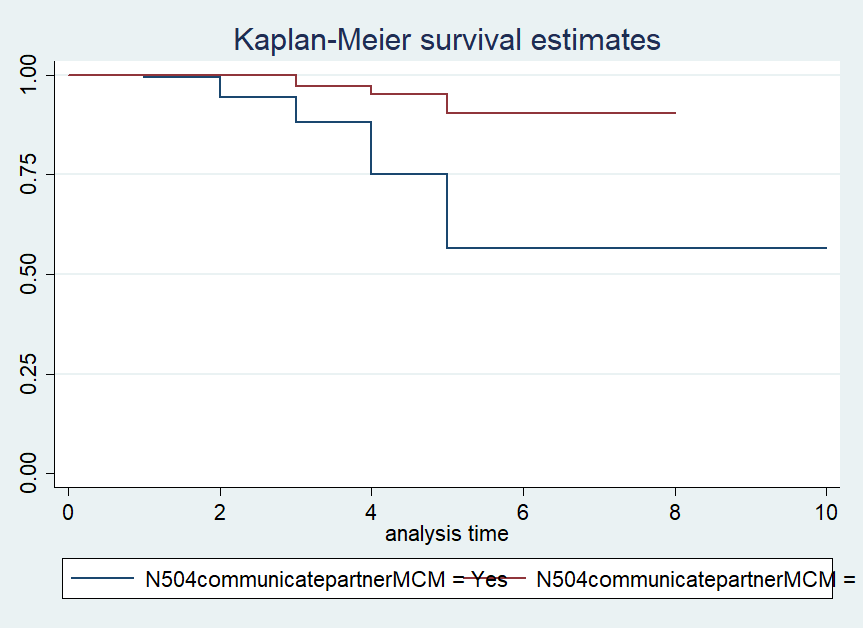


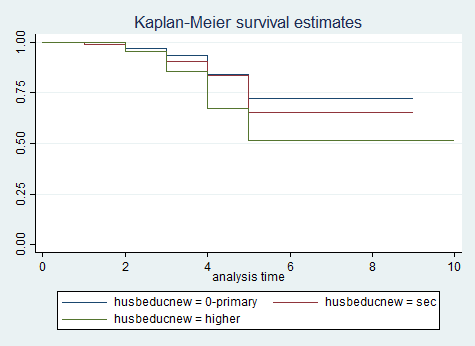


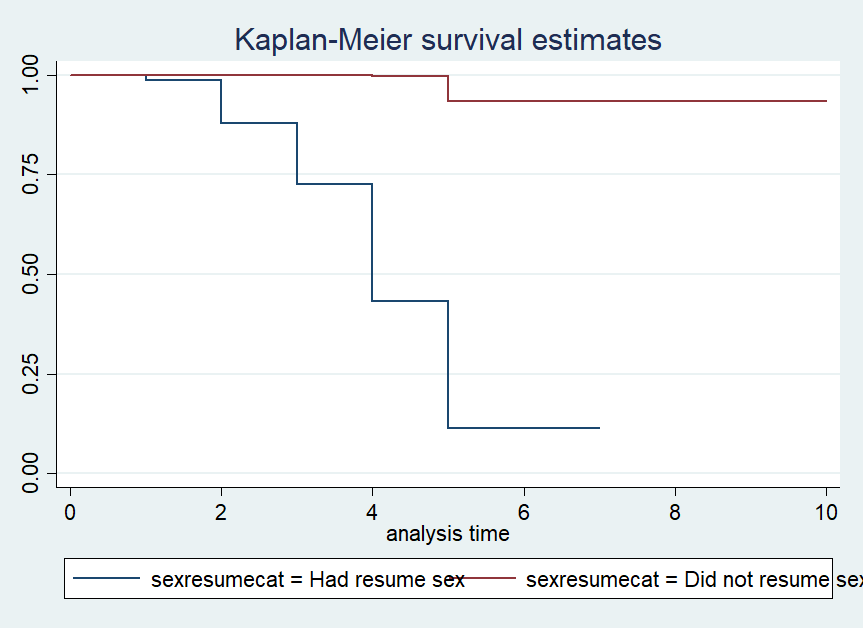

Supplement: Supplementary file 1 — Supplementary Material 1 [file 12905_2023_2809_MOESM1_ESM.doc]
