# Supplementary material for: Time to initiation of modern contraceptive method use after childbirth and its predictors in Southern Ethiopia: a retrospective follow-up study
Source: BMC Womens Health. 2023 Dec 8;23:658. doi: 10.1186/s12905-023-02809-y (PMC10704612; doi:10.1186/s12905-023-02809-y)
Supplement: Supplementary file 2 — Supplementary Material 2 [file 12905_2023_2809_MOESM2_ESM.doc]

**Additional file 1. Test of proportional-hazards assumption**

| **Variables** | **rho** | **Chi2** | **df** | **Prob>chi2** |
| --- | --- | --- | --- | --- |
| Maternal age | -0.11070 | 1.87 | 1 | 0.1712 |
| Residence | -0.02189 | 0.08 | 1 | 0.7800 |
| Maternal education | 0.04330 | 0.27 | 1 | 0.6064 |
| Husband/partner education | 0.06381 | 0.71 | 1 | 0.4001 |
| Parity | 0.14534 | 3.46 | 1 | 0.0631 |
| Number of alive children | 0.04303 | 0.28 | 1 | 0.5951 |
| Family planning counseling during antenatal care visits | -0.11481 | 2.02 | 1 | 0.1554 |
| Future reproductive plan | 0.02038 | 0.06 | 1 | 0.8028 |
| Communication with husband/partner about modern contraceptive methods | 0.02966 | 0.15 | 1 | 0.6972 |
| Husband approval using modern contraceptive methods | -0.02384 | 0.09 | 1 | 0.7621 |
| Decision-maker for modern contraceptive method use | -0.08116 | 1.07 | 1 | 0.3013 |
| Return of menses | -0.40036 | 27.61 | 1 | 0.0000 |
| Resumption of sexual intercourse after childbirth | 0.31690 | 17.78 | 1 | 0.0000 |
| **Global test** |  | **45.08** | **13** | **0.0000** |
